# Characteristics of the Waste Wood Biomass and Its Effect on the Properties of Wood Sanding Dust/Recycled PP Composite

**DOI:** 10.3390/polym14030468

**Published:** 2022-01-24

**Authors:** Sanita Vitolina, Galia Shulga, Brigita Neiberte, Jevgenijs Jaunslavietis, Anrijs Verovkins, Talrits Betkers

**Affiliations:** Latvian State Institute of Wood Chemistry, Dzerbenes 27, LV-1006 Riga, Latvia; galshulga@inbox.lv (G.S.); brigita.neiberte@kki.lv (B.N.); jevgenijs.jaunslavietis@kki.lv (J.J.); anrijs.verovkins@kki.lv (A.V.); betalrits@gmail.com (T.B.)

**Keywords:** waste wood biomass, wood–plastic composite, hybrid filler, coagulation, wood processing wastewater

## Abstract

To decrease climate changes, more research focuses on decreasing waste wood biomass (WWB) burning and increasing its conversion into value-added products. The WWB was isolated from model wood processing wastewater with a new hybrid coagulant by the coagulation/flocculation method. This study is aimed to characterize the WWB and to investigate its effect in the composition of a hybrid lignocellulosic filler on the properties of recycled polypropylene (rPP)-based wood–plastic composites (WPCs). The waste biomass contained high-molecular lignin and hemicelluloses substances and represented a finely dispersed powder. It was hydrophobic and was characterized by enhanced thermal stability. To minimize the negative effect of polymer wastes on the environment, recycled polypropylene as a polymer matrix was used with the hybrid filler in fabricating WPC samples. The presence of the coagulated WWB in the hybrid filler composition positively affects the mechanical properties, water uptake and dimensional stability of the composite samples. Such a behavior of the waste biomass showed its function as a compatibilizer, which promoted the interfacial adhesion in the composite system.

## 1. Introduction

Climate instability, land degradation, water scarcity and carbon-intensive energy systems are just some of the socio-environmental challenges the world is facing today. Nowadays, to achieve socially and environmentally friendly climate and energy policies, waste wood biomass research focuses on decreasing its burning and increasing its conversion into value-added products. Waste wood biomass in the form of wood sawdust, bark, chips shavings, wood sanding dust, etc., representing forestry and wood mechanical processing side streams, plays a significant role as raw materials in the European bioeconomy [1].

Wood–plastic composites (WPCs) are a young generation of (semi)-biocomposites that have attracted increasing research interest in both scientific and industrial areas over the past few decades [2,3]. The use of WPCs is rapidly growing worldwide in the automobile industry and building engineering for the production of consumer goods, domestic and technical materials, etc. Such a type of composite material combines the advantages of both synthetic polymers and lignocellulosic fillers into one whole. The low processing costs of the polymers, their flexibility and easiness of molding enable their application as a polymer matrix in WPCs. Polyolefins (polyethylene, polypropylene) [4,5,6,7], polylactic acid [8,9] and polyvinyl chloride [10], which have a high melting flow index and low softening temperatures, are usually mixed with lignocellulosic materials for fabricating WPCs. To minimize the negative effect of synthetic polymers on the environment, their re-use as a polymer matrix in WPCs is a promising practice for rational recovering of polymer wastes [5,6,7,9,11,12,13,14,15]. It was shown that, in some cases, the mechanical properties of the WPCs based on recycled polyolefins were equal or higher than those of the WPCs based on virgin polymers [16]. Polypropylene (PP) is one of the most commonly used thermoplastic polymers due to its good physical, mechanical, thermal and chemical properties. Recycled PP (rPP), produced from different polypropylene wastes, is often used in fabricating WPCs [5,6,7,12].

The utilization of lignocellulosic materials as a filler provides a low cost, biodegradability, light mass, enhanced filling degree and decreased abrasiveness of WPCs [2,17]. There are two types of lignocellulosic fillers, namely fibers and particles. The particles have a shape that could be approximated by a round form. The fibers have an aspect ratio (ratio of the length to diameter) of much more than unity. Wood fibers, for example, wood flour, represent the most widely used short fibers, which occupy an intermediate place in size between particulates and continuous fibers. Agro-based lignocellulosic fibers derived from flax, hemp, sisal, kenaf, wheat straw, jute, sugar cane bagasse, etc., are commonly used in WPCs in the form of continuous fibers.

Another representative of the particulate filler could be waste wood biomass (WWB) from wood processing wastewater, which contains lignin and hemicelluloses substances. This biomass represents solid wastewater sludge that is formed as a result of wastewater treatment with organic or inorganic reagents in the coagulation/flocculation process at wood processing plants [18,19,20]. In industrial veneer production in eastern Europe, it is common to soak logs at elevated temperatures prior to peeling. This hydrothermal pre-treatment is carried out to soften the log and makes cutting easier during the peeling process [21]. The treatment of wood with water at elevated temperatures results in physical and chemical changes in wood cell walls and the dissolution of the formed products of wood hydrolysis. As a result, water is polluted with both suspended and dissolved wood origin substances (hemicelluloses, lignin, extractives, various destruction products, such as carboxylic acids, aldehydes and ketones), which are responsible for the relatively high level of chemical oxygen demand (up to 7000 mg L^−1^) [22,23]. The WWB obtained as a result of the water purification with reagents is traditionally used for energy recovery by its combustion [24]. At the same time, WWB represents a large raw resource of renewable organic substances to obtain value-added products. Due to the biomass chemical composition, surface activity and cation exchange capacity, WWB has been offered for applications in the production of construction materials [25,26], sorbents [27,28], for soil structuring [29] and as a fertilizer [30,31,32].

It is known that, for fabricating WPCs, a mixture of a lignocellulosic filler with a polymer matrix must be heated to the above polymer’s melting point, providing sufficient shearing agitation to ensure the complete mixing of fibers with the polymer matrix. The following fabrication methods of WPCs, such as extrusion molding, injection molding, compression molding, hot press molding and hand lay-up, are described in detail in the literature [33,34,35]. In recent years, 3D printing has been used for the preparation of WPCs as a newer and advanced fabrication mode [36].

The performance and behavior of WPCs critically depend on the effectiveness of the load/stress transfer across the interface. In turn, this effectiveness is fully determined by the compatibility between the hydrophobic polymer matrix and the hydrophilic lignocellulosic filler. The improvement of the adhesion affinity between polyolefins and lignocellulosic fibers in WPCs can be achieved by both the activation/functionalization of the fiber surface and/or the application of specific functional additives, such as compatibilizers, coupling agents or adhesion enhancers [37,38]. There are various chemical methods for the activation and functionalization of lignocellulosic fibers as a filler, namely mercerization, acetylation, benzylation, graft copolymerization, treatment with acids, peroxide, various anhydrides, permanganate, silane, etc., which aim to increase the physicochemical interaction between the polymer matrix and the lignocellulosic filler in WPCs [39,40].

The variability in the chemical composition and properties of lignocellulosic fillers due to their different origins allows combining lignocellulosic fibers of different wood species, agro-based plants and other biomass into a hybrid filler for fabricating WPCs with improved exploitation properties [41,42,43,44,45]. If wood is mixed with cheaper lignocellulosic materials, the reduction in the WPC price can be essential.

The aim of the present work was to characterize the solid waste wood biomass isolated from the wood processing wastewater and study its effect in a composition of the hybrid lignocellulosic filler on the properties of recycled polymer-based wood–plastic composites.

## 2. Materials and Methods

### 2.1. Materials

Birch wood sanding dust, a waste of the mechanical treatment of plywood, was supplied by a Latvian enterprise. According to the results of its fractionation, the birch wood sanding dust had the following particle sizes and their content: less 250 μk—33%, 250−500 μk—58% and more 500 μk—9%. The wood dust was characterized by elemental analysis (Elementar Analysensysteme GmbH, Langenselbold, Germany), and the wood component composition was characterized according to Klason and Kürschner chemical procedures for lignin [46] and cellulose [47], respectively, as well as hemicelluloses [48]. The found elemental composition of the wood residue was the following: 46.83% C, 6.88% H, 45.90% O, 0.28% N and 0.11% S. The content of cellulose, lignin and hemicelluloses in the wood dust was 48.7%, 21.8% and 29.5%, respectively.

Recycled polypropylene (rPP) (density: 0.9 t/m^3^, melt flow index: 5.2 g/10 min at 230 °C and 2.16 kg) was supplied by a Latvian polymer recycling plant (Nordic Plast Ltd., Olaine, Latvia) and used as a thermoplastic polymer matrix. Its powder was obtained by milling the granules in a knife mill using a sieve with a size of less than 500 µk.

#### 2.1.1. Wood Processing Wastewater

To obtain WWB, the model wastewater, simulating the wastewater of the hydrothermal treatment of birch wood in open water basins of plywood plants, was used. The wastewater was obtained by hydrothermal treatment of birch wood sawdust with 0.01 M NaOH at a hydromodulus of 1/50 (the mass ratio of the air-dried sawdust to water) and a temperature of 90 °C for 4 h with the followed filtration. The main parameters of the model wastewater are listed in Table 1.

The zeta potential value of the model wastewater was −30 mV, indicating its high colloidal stability due to the presence of various thermal hydrolyzed wood products with the negatively charged groups.

#### 2.1.2. Determination of Composition of the Model Wastewater

**Figure 1:**
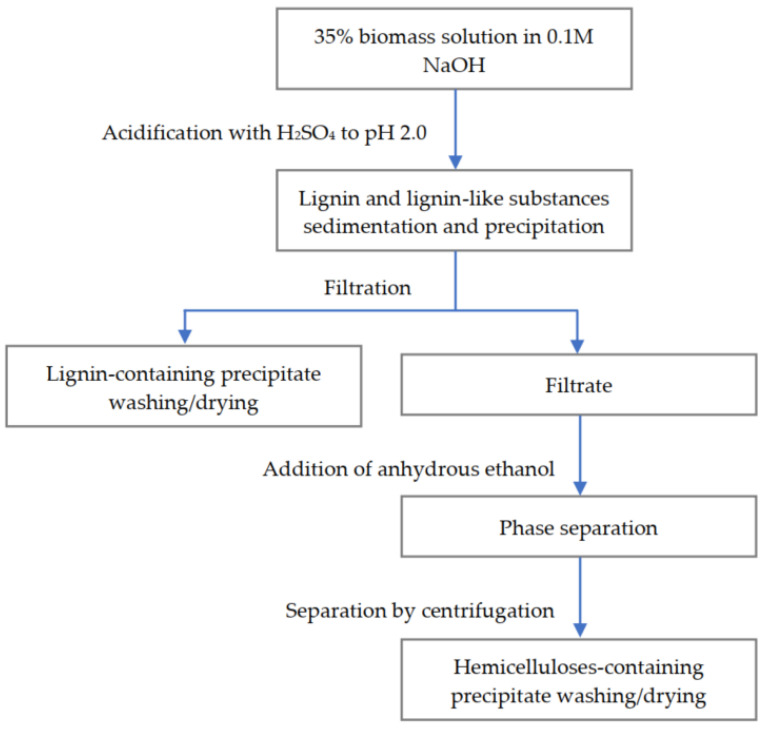
Scheme of model wastewater fractionation.

To determine the composition of the model wastewater in terms of wood components, the developed method [49] was employed. The scheme of the applied fractionation is given in Figure 1. The biomass sample was obtained by ordinary evaporation of the model wastewater by drying, at first, at room temperature and then at 60 °C in an oven was obtained. To prepare a 35% WWB solution, it was dissolved in a 0.1 M NaOH solution. Then, the WWB water concentrate was, at first, acidified with 20% sulfuric acid to a pH of 2.0 at room temperature, with the following filtration and centrifugation of the obtained suspension for separating the lignin-containing fraction. The lignin precipitate was washed with distilled water to pH 5.5 and dried in an oven at 40 °C. Anhydrous ethanol was added to the obtained filtrate at the volumetric ratio of ethanol/filtrate close to 4. After the addition of anhydrous ethanol, the hemicelluloses-containing fraction precipitated and then was isolated by centrifugation, washed with the ethanol and dried in an oven at 40 °C. The content of hemicelluloses and lignin-containing fractions in the biomass was assessed from the masses of the obtained dried precipitates.

#### 2.1.3. Separation of Waste Wood Biomass by the Coagulation/Flocculation Process

The separation of WWB was performed by the coagulation/flocculation method using the developed hybrid coagulant [50], representing the polymer complex of the composite coagulant based on polyaluminium chloride [51] and high-molecular polyethylenimine. Owing to the hybrid nature of the developed coagulant comprising the coagulation/flocculation properties of the inorganic coagulant and the organic flocculant, it was characterized by the high removal efficiency of the WWB from the model wastewater (almost 97%) compared to the initial constituents.

#### 2.1.4. Activation of Wood Sanding Dust

The activation of the wood sanding dust was carried out by alkaline treatment using a 5 L three-neck flask equipped with a return condenser, a thermometer and a stirrer under the following conditions: a NaOH concentration of 0.025 g/dL, temperature of 60 °C and duration of 5 h at a hydromodulus (dust/water mass ratio) of 1/20. The activated wood sanding dust was washed to a neutral medium, dried and milled with a planetary ball mill (Retsch, Haan, Germany) for 15 min at 300 rpm. After sieving with a Vibratory micromill called PULVERISETTE 0 (Frisch GmbH, Idar-Oberstein, Germany), a fraction of the wood particles less than 100 μm was used for obtaining WPCs. The relative content of cellulose, lignin and hemicelluloses in the activated birch wood microparticles was 55.7%, 22.7% and 21.6%, respectively. The activated wood sanding dust < 100 µk, together with the WWB microparticles, was used in the composition of the hybrid filler for fabricating WPC samples.

#### 2.1.5. Preparation of Wood–Plastic Samples

Before fabricating the WPC samples, the powdered rPP with the mixture of the activated birch wood dust and the WWB microparticles was mixed for 5 min at room temperature with a vibratory micromill PULVERISETTE 0 (Frisch GmbH, Idar-Oberstein, Germany). The samples for tensile and bending tests were prepared by the extrusion and molding methods using HAAKE MiniLab II and MiniJet II (Thermo Fisher Scientific, Karlsruhe, Germany). The processing conditions were as follows: a chamber temperature of 175 °C, screw rotation speed of 130 rpm, circulation time in the extruder of 5 min, injection pressure of 60 MPa and mold temperature of 120 °C. The WPC samples for tensile and bending tests were fabricated according to ASTM D638 (2007) and ISO 178 (2010), respectively. In a three-point bending test, samples with a length of 80.0 mm, width of 10.0 mm and thickness of 4.0 mm were used. “Dogbone” samples for a tensile test have the following sizes: overall length of 63.0 mm, overall width of 9.4 mm, length of narrow section of 9.4 mm, width of narrow section of 3.15 and thickness of 3.15 mm. The filling degree in the WPC samples was 30 %. The compositions of the WPC samples are given in Table 2.

### 2.2. Methods

#### 2.2.1. Elemental Analysis

The elemental analyzer Vario MACRO CHNS (Elementar Analysensysteme GmbH, Langenselbold, Germany) with a heat conduction detector was used to determine the elemental composition (C, H, N, S, O) of the biomass samples. The determination of the metal content in the waste wood biomass was performed in accordance with ISO 15586:2003 [52].

#### 2.2.2. Pyrolysis-Gas Chromatography-Mass Spectrometry (Py-GC/MS) Analysis

For analytical Py-GC/MS analysis, a Double-shot Pyrolyzer Py-2020iD (Frontier Lab Ltd., Cape Town, South Africa) with a GC/MS-QP2010 gas chromatograph-mass spectrometer (Shimadzu, Kyoto, Japan) was used.

#### 2.2.3. Fourier Transform Infrared (FT-IR) Spectroscopy

The FT-IR study was performed using a Perkin Elmer Spectrum One apparatus (Perkin Elmer, Waltham, MA, USA) at the range of wavenumbers from 4000 to 450 cm^−1^ (30 scans), at a resolution of 4 cm^−1^. The tablets were prepared by mixing 20 mg of a sample with 200 mg of KBr, and then the tablet compression method was used.

#### 2.2.4. Thermogravimetric Analysis (TGA)

To evaluate the thermal stability of WWB, thermogravimetric analysis (TGA) was performed using a Mettler Toledo TGA/SDTA 851e instrument (Mettler Toledo, Columbus, OH, USA) in the temperature range from 25 to 600 °C with the heating rate of 5 °C min^−1^.

#### 2.2.5. Particle Size

To determine the sizes of the air-dried waste wood biomass, the Laser Particle Sizer ANALYSETTE 22 NanoTec (Fritsch GmbH, Idar-Oberstein, Germany) with a measuring range of 10 nm–1 mm and Leica MZ 16 A stereomicroscope (Leica Microsystems Ltd., Milton Keynes, UK) were applied.

#### 2.2.6. Surface Free Energy and Its Energetic Components

To study the surface properties, WPC samples with dimensions of 60 × 10 × 1 mm^3^ were prepared using HAAKE MiniLab II and MiniJet II (Thermo Fisher Scientific, Karlsruhe, Germany). Before testing, the samples were conditioned at 60 °C in a drying cabinet for 24 h and then stored for 1 h at room temperature in a desiccator. The total surface free energy (SFE) and its dispersive (Lifshitz–Van der Waals interactions) and polar (Lewis acid-base interactions) components were calculated using the Owens–Wendt–Rabel–Kaelble method [53], for which the contact angle values with different liquids, namely water, DMSO and diiodomethane, were measured. The contact angles were measured with a Kruss tensiometer K 100 M (Kruss GmbH, Hamburg, Germany) using the Wilhelmy method. For the calculation of SFE, a special software program (Advance Software Assurance, version 3.2), mounted in the tensiometer, was applied. Five replicates were tested for each sample, and the standard deviation for each value was found.

#### 2.2.7. Water Uptake and Swelling

The water sorption capacity and swelling degree of the WPC samples were determined according to ASTM D 570 [54]. The WPC samples had been previously dried in an oven (60 °C, 24 h) and then were immersed in a desiccator with distilled water. At regular intervals, the samples were removed, patted dry and weighed. The thickness changes were measured with a caliper. The water absorption (WA) and dimensional swelling in thickness (TS) were determined by the following formulas:

WA (%) = (WA_t_ − WA_o_)/WA_o_ × 100 (1)

TS (%) = (TS_t_ − TS_o_)/TS_o_ × 100 (2)

where WA_t_ and WA_o_ are the samples mass at the certain and initial times, respectively, and TS_t_ and TS_o_ are the sample thickness at the certain and initial times, respectively. Five replicates were tested for each sample, and the standard deviation for each value was found.

#### 2.2.8. Mechanical Tests

Mechanical properties were determined with a universal machine “Zwick” (Zwick/Roell, Ulm, Germany) with a load capacity of 0.5 kN at a rate of 50 mm/min and 2 mm/min for tensile and bending tests according to ASTM D638 (2007) and ISO 178 (2010), respectively, with the help of the software program (TestXpert, Zwick/Roell, Ulm, Germany). The samples were conditioned at 60 °C for 24 h and then placed in a desiccator before testing. Five replicates were made for each mechanical testing, and the standard deviation for each index was found.

#### 2.2.9. Scanning Electron Microscopy (SEM)

The morphology of the obtained WPC samples was examined using a Tescan Vega TS5136 scanning electron microscope (Tescan, Brno, Czech Republic).

#### 2.2.10. Milling

The milling of the activated wood sandling dust was carried out with a planetary ball mill (Retsch, Haan, Germany). To fractionate the milled wood dust, a mill “Pulverisette 0” (Frisch GmbH, Idar-Oberstein, Germany) with a set of sieves was used.

## 3. Results and Discussion

### 3.1. Characterization of Waste Wood Biomass Samples

**Figure 2:**
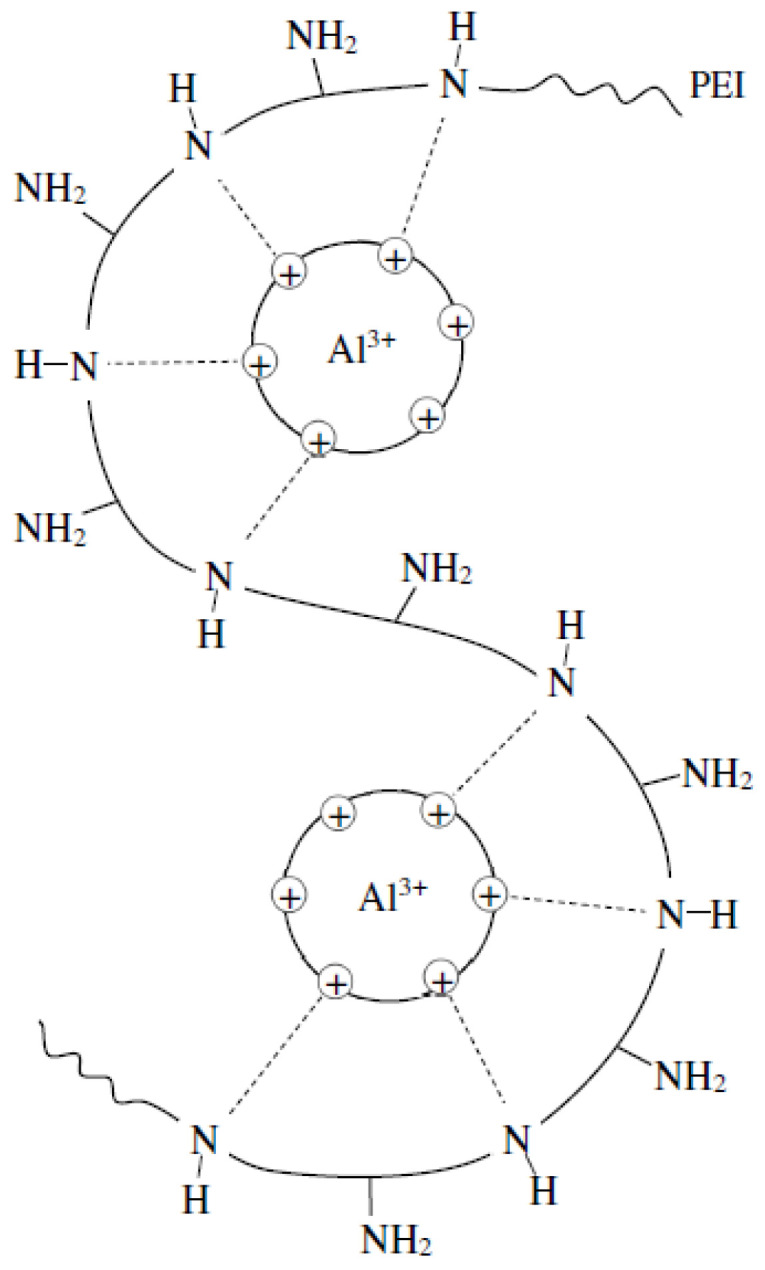
Schematic image of the hybrid coagulant.

**Figure 3:**
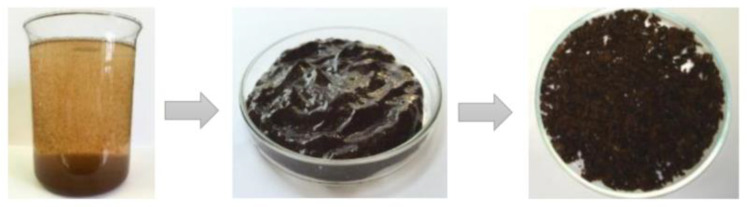
The waste wood biomass powder obtained from the coagulated wastewater by centrifugation followed by drying.

In the work, two samples of WWB were studied. The first sample was isolated from the wastewater by its evaporation at room temperature first and then at 60 °C for 48 h. The results of the fractionation of the evaporated WWB showed that the evaporated WWB consisted of three main fractions, namely hemicelluloses (75.2%), lignin-containing substances (13.5%) and the low molecular products (11.3%) of the wood thermal hydrolysis. The second sample was extracted by the coagulation/flocculation process of the wastewater with the developed hybrid coagulant representing the polymer complex of the composite coagulant based on polyaluminium chloride and high-molecular polyethylenimine [50]. The schematic image of the developed coagulant is given in Figure 2. The coagulated WWB after centrifugation represented a pasty mass with a moisture content of about 92–93%, but after its drying, it was a finely dispersed brown powder (Figure 3).

The obtained WWB samples differed significantly in water solubility. If the evaporated WWB sample was soluble in water, the coagulated WWB was hydrophobic. The solubility of the samples depended on the method of their isolation from the model solution. The evaporated WWB was obtained by evaporation of the model solution, first in a water bath, then in a crystallizer at room temperature, followed by drying in an oven at 60 °C to an air-dry state. During this procedure, hemicelluloses, lignin and the low-molecular-mass products of the wood thermal hydrolysis did not change their solubility in water. The coagulated wood biomass was obtained by its precipitation from the model wastewater with the developed coagulant due to electrostatic and hydrophobic interactions with followed drying of the obtained coagulate at 60 °C to an air-dry state. The average elemental composition of the WWB samples is shown in Table 3.

The biomass enhanced ratio of O/C in both biomass samples could indicate the presence of a high number of oxygen-containing groups, including hydroxyl, carbonyl and carboxyl groups in the biomass samples. The coagulated biomass, compared to the evaporated WWB sample, also contains aluminum and nitrogen that testifies the presence of the hybrid coagulant in its composition. The elemental composition of the coagulated WWB sample shows a decreased content of carbon and oxygen compared to the case of the evaporated WWB sample, which could be related to the lower content of the low-molecular aromatic substances in the coagulated biomass sample and the higher content of hemicelluloses therein.

**Figure 4:**
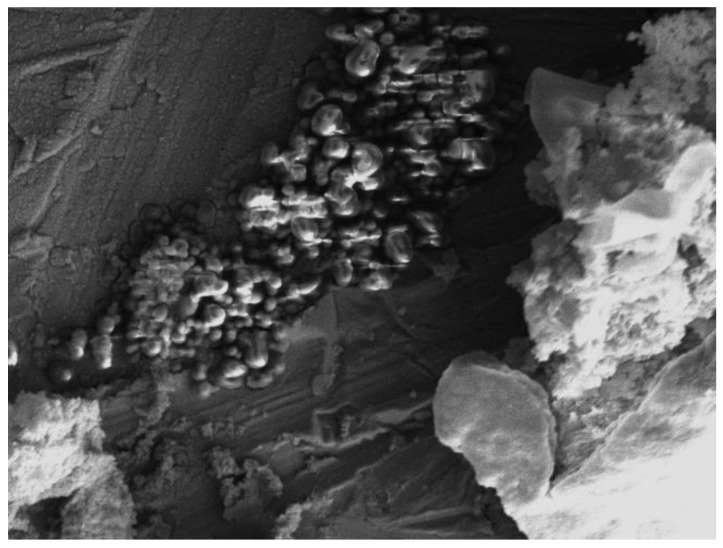
SEM image of the coagulated waste wood biomass (×2000).

The image of coagulated WWB, produced by scanning its surface, is given in Figure 4. This image shows the presence of coagulated lignin- and hemicelluloses-containing particles in the biomass sample.

**Figure 5:**
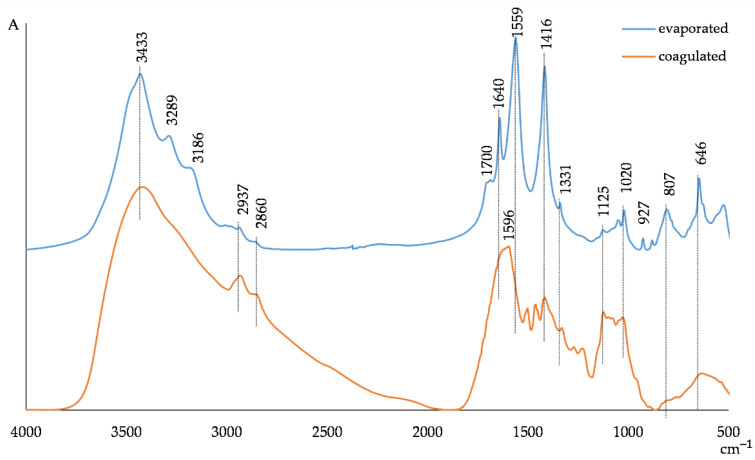
FT-IR spectra of evaporated and coagulated waste wood biomass samples.

For the characterization of the chemical composition of the biomass samples, FT-IR spectroscopy was used. The interpretation of the obtained results was based on the literature data concerning the studies of lignin and hemicelluloses by this method [55,56,57]. According to the FT-IR spectra of the biomass samples illustrated in Figure 5 and the assignments of the main absorptions shown in Table 4, the strong band in the region of 3300–3500 cm^−1^ is attributed to both aromatic and aliphatic hydroxyl groups of wood origin products, including hemicelluloses, lignin-containing substances and low-molecular products of the wood thermal hydrolysis.

The bands at 3289 and 3186 cm^−1^ associated with C–H stretch vibrations in the low-molecular wood thermal hydrolysis products are in the spectrum of the evaporated WWB and absent in the FT-IR spectrum of the coagulated biomass. Two bands with peaks at 2937 and 2860 cm^−1^, arising from C–H stretching vibrations in methoxyl, methyl and methylene groups in the aromatic and saccharide structures, are shown in the spectrum of the coagulated WWB, and these bands are essentially less intensive for the evaporated biomass sample. The more pronounced diffusion character of the FT-IR spectrum of the coagulated WWB sample and its higher intensity, compared to the case of the FT-IR spectrum of the evaporated biomass in this absorbance region, could indicate a well-developed network of hydrogen bonds between the oxygen-containing functional groups of the lignin-containing and hemicelluloses substances with amine groups in the coagulated biomass.

The narrow, strong bands at 1700, 1640 and 1559 cm^−1^ in the evaporated biomass FT-IR spectrum may be assigned to the presence of ketones, carbonyls and ester groups, both in hemicelluloses and lignin-containing substances, as well as in the wood hydrolyzed low-molecular products. At the same time, the spectrum of the coagulated WWB shows a broad diffusion band in the region of 1710–1540 cm^−1^ with a maximum at 1596 cm^−1^ instead of the above-indicated peaks. This broad band is complex and could involve the vibrations of the oxygen-containing groups of lignin and hemicelluloses bonded with the amine groups of the hybrid coagulant. It is known that amine groups absorb in the regions of 1650–1580 cm^−1^ (N-H bending vibrations) and 3000–2800 cm^−1^ (N–H stretching vibrations). The peaks at 1596 and 1501 cm^−1^ in the FT-IR spectrum of the coagulated biomass sample could also correspond to the overlapped aromatic skeletal vibrations of the lignin structures, indicating that the hybrid coagulant binds predominantly high-molecular lignin fragments from the wastewater. This also confirms comparatively more intensive peaks at 2937 and 2860 cm^−1^ arising from C–H stretching vibrations in methoxyl groups in the FT-IR spectrum of the coagulated biomass relative to the spectrum of the evaporated WWB. At the same time, the FT-IR spectrum of the evaporated WWB sample shows the absorbance at 1416 and 1331 cm^−1^, assigned to aromatic skeleton vibrations and syringyl ring breathing with C=O stretching, could reflect the presence of a whole set of aromatic substances with different molecular masses in this sample. The bands in the region of 1125–800 cm^−1^ are typical for hemicelluloses. This region contains C–C ring vibrations, overlapped with the stretching vibrations of C–OH side groups and the C–O–C glucosidic band vibrations. The intensity of absorbance bands in the region of 1125–1020 cm^−1^ in the FT-IR spectrum of the coagulated biomass is higher than that of these bands for the evaporated WWB sample. The presence of the peaks at 927 and 807 cm^−1^ associated with C–H bending vibrations in the FT-IR spectrum of the evaporated biomass sample and their absence in the spectrum of the coagulated biomass could be related to the presence of the wood’s low-molecular products owing to its thermal hydrolysis. Thus, the coagulated WWB contains mainly the high-molecular lignin-containing and hemicelluloses substances, unlike the evaporated biomass sample involving the wood hydrolysis products with a wide scale of molecular masses.

**Figure 6:**
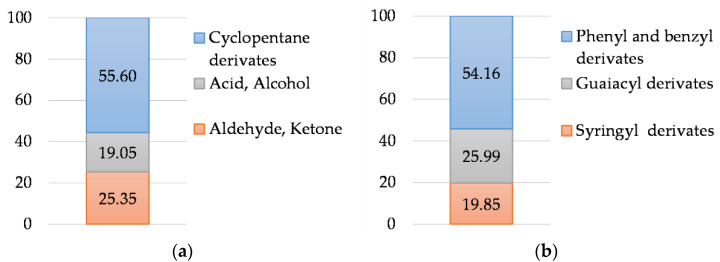
Decomposition products of (**a**) hemicelluloses and (**b**) lignin components of coagulated waste wood biomass studied by Py-GC/MS.

The study of the chemical composition of the coagulated WWB sample by Py-GC/MS (Figure 6) indicates that the hemicelluloses substances during the pyrolysis form 55.60% of cyclopentane derivatives, 19.05% of organic acids and alcohols and 25.35% of aldehydes and ketones.

The lignin component of the biomass decomposes into phenyl and benzyl derivatives (54.16%), guaiacyl (25.99%) and syringyl (19.85%) low-molecular substances. No toxic decomposition products have been identified.

**Figure 7:**
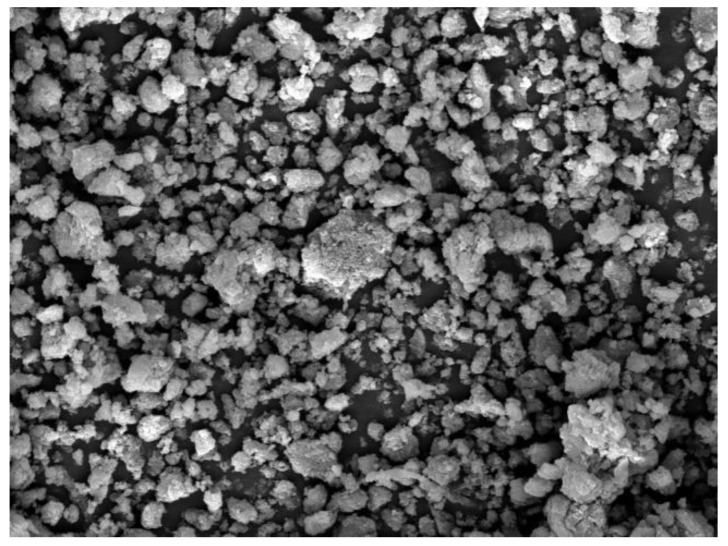
SEM image of the coagulated waste wood biomass sample (×2000).

The granulometric analysis of the coagulated WWB sample, carried out with a laser particle sizer, was characterized by the wide distribution of particle sizes varied from 3 to 50 μk. More than 50% of the biomass particles were smaller than 10 µk. The SEM image of the coagulated WWB sample (Figure 7) demonstrates the finely dispersed nature of the air-dried coagulated biomass sample.

**Figure 8:**
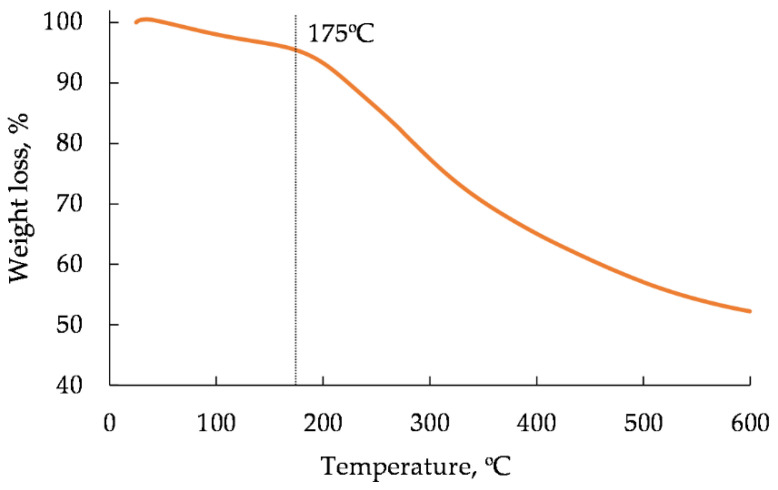
Thermogravimetry curve of air-dried coagulated waste wood biomass sample.

It is known that the thermal stability of a lignocellulosic filler is a basic condition for its processing to fabricate WPCs. According to Figure 8, the mass loss of the coagulated WWB at 175 °C does not exceed 4.5%. Because the values of a thermal degradation temperature of hemicelluloses and lignin are higher than 200 °C, the mass loss of the coagulated WWB at 175 °C is mainly associated with the dehydration of the biomass sample. Above 200 °C, a more rapid mass loss of the biomass sample is observed that indicates its thermal decomposition.

### 3.2. Effect of Biomass on the Properties of Wood–Plastic Composite

**Figure 9:**
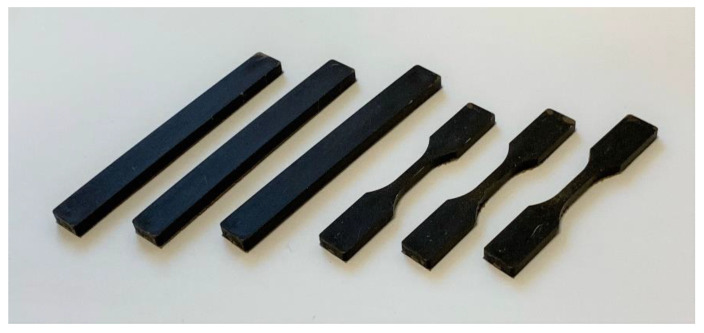
The obtained wood–plastic composites for mechanical tests.

The composite raw blends were prepared by mixing microparticles of the rPP, the activated birch wood sanding dust and the coagulated WWB sample in the vibratory micromill for 5 min. Then, the composite samples with rPP for tensile and bending tests were prepared by the extrusion and molding methods. The photo of the fabricated WPC samples for tensile and bending tests is shown in Figure 9.

The mechanical properties of the rPP-based composite samples containing the hybrid filler with different content of the coagulated biomass with a filling degree of 30% are presented in Table 5. For comparison, the mechanical properties of the WPC samples filled with the initial and activated sanding dust without the biomass are also shown here.

It can be seen that the mechanical properties of the obtained WPC sample filled with the activated microparticles are much higher than those of the samples filled with the initial wood dust. It is known that the alkaline treatment of lignocellulosic fibers is one of the most commonly used chemical methods for improving interface adhesion in WPCs. This is an environmentally friendly, effective method of modifying wood fibers, which does not require the use of special equipment and is relatively cheap [58,59,60]. During this treatment, the destruction of hydrogen bonding in the lignocellulosic matrix network occurs. This leads to the increase in the amorphous cellulose content and surface roughness of lignocellulosic fibers and also causes the disintegration of bundles of the fibers, reducing their sizes. This favors the interface adhesion between a polymer matrix and a lignocellulosic filler. Lower indicators of the mechanical properties for the WPC sample with the untreated wood dust are attributed to the insufficient compatibility between the rPP and the wood dust particles.

**Figure 10:**
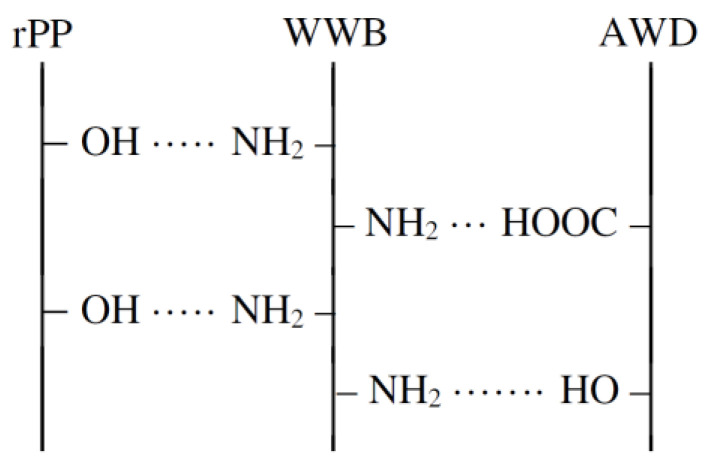
Scheme of interaction of waste wood biomass with activated wood dust and recycled polypropylene.

According to Table 5, the presence of the biomass sample in the WPC composition leads to an increase in the mechanical properties of the composite. With increasing the biomass content in the WPC sample from 1% to 5%, its tensile and bending strength increases by 17% and 21%, Young’s and bending modulus by 15% and 23% and tensile and bending deformation decreases by 12% and 18%, respectively, relative to the mechanical properties of the biomass-free composite sample with the activated wood dust filler. At the same time, the hybrid filler containing 1% of WWB (based on the mass of the composite) does not significantly affect the mechanical properties of the WPC sample since their increase relative to the same indicators of the WPC samples with activated wood dust does not exceed 1–4%. With an increase in the WWB content in the WPC sample of more than 5%, a decrease in its positive effect on the mechanical properties of the composite occurs, and the values of the tensile and bending strengths, as well as the strength modulus at the WWB content of 10%, become equal to those of the composite sample containing 1% of WWB. Such behavior of the composite material in the presence of the hybrid filler may be due to the structural features of the coagulated biomass. Since the surface of the coagulated biomass contains both completely hydrophobic regions, which are formed as a result of the interaction of the biomass components with the hybrid coagulant, and free functional groups (carboxyl-, hydroxyl-, amino-), which are located in the defected regions (e.g., tails, loops), the coagulated biomass particles have to exhibit adhesive properties. It was supposed that the biomass particles could interact during the processing of the WPC samples both with the wood dust surface due to physicochemical interactions, including Van der Waals forces and hydrogen bonds, and with recycled polypropylene surface, taking into account the presence of oxygen-containing groups on it. With the increase in the biomass content in the composition of the hybrid filler, its contribution to enhancing the interface interaction with the formation of structured boundary layers in the WPC samples grows. This behavior of the biomass in the composite is similar to the effect of a compatibilizer, which increases the compatibility of the hydrophilic filler with the hydrophobic polymer matrix at the interface. Similar to our case, the excess of the content of compatibilizers in composite systems leads to the deterioration of the mechanical properties of WPCs [61]. A scheme of the possible interaction of the WWB with the activated wood dust (AWD) and rPP is shown in Figure 10.

It is known that the ability of WPCs to uptake water vapors during exploitation can negatively affect their mechanical strength, dimensional stability and resistance against biological decay [62,63,64]. Using the Wilhelmy method, water contact angles were found for the WPC samples with the activated wood dust and the hybrid filler with the different content of the WWB sample. The contact angle of the sample without the biomass was 89.1 ± 1.1°, while, for the samples with the hybrid filler, the contact angle was enhanced from 89.8 ± 1.5° to 93.2 ± 1.4° when the biomass content increased from 1% to 5%. The increased water contact angles for the WPC samples in the presence of the biomass showed that the surface of these composites attracts less water, which could positively affect their exploitation long term properties. To confirm this assumption, the moisture uptake and dimensional swelling of the obtained composite sample were investigated.

**Figure 11:**
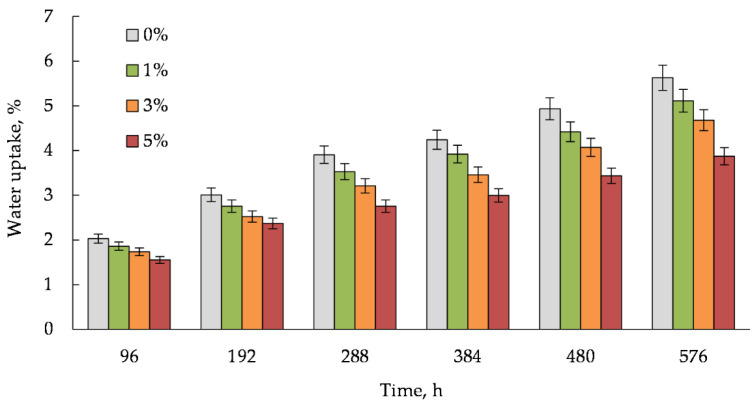
Water uptake of wood–plastic composite samples filled with the hybrid filler. The percentage indicates the content of the waste wood biomass in the samples.

**Figure 12:**
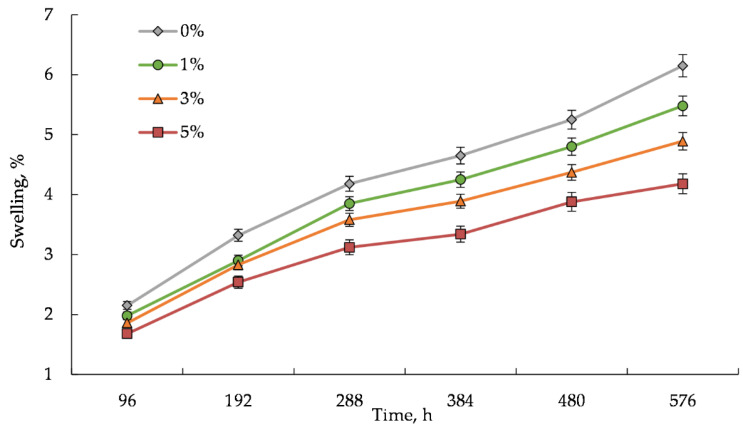
Swelling degree of wood–plastic composite samples filled with the hybrid filler. The percentage indicates the content of the waste wood biomass in the samples.

Figure 11 and Figure 12 demonstrate the results of the study of the water sorption and swelling degree of the composites, which were obtained for 96, 192, 288, 384, 480 and 575 h. For this purpose, the WPC samples were totally immersed in distilled water at room temperature (23 °C).

It can be seen that the sorption and swelling degree of the WPC samples with the hybrid filler are lower than those of the sample with the activated wood dust. The higher content of the WWB in the hybrid filler, the higher decrease in both the water uptake and swelling of the WPC sample. According to the obtained results (Figure 11 and Figure 12), with increasing the biomass content in the WPC from 1% to 5%, its water uptake decreases by 31% and 45% but its swelling capacity by 28% and 47%, respectively, relative to these indicators of the biomass-free composite sample with the activated wood dust, immersed for 96 and 576 h. It is known that the water uptake and dimensional swelling of WPCs are related to the compatibility between a polymer matrix and a filler, which in turn is determined by the degree of hydrophilicity of the lignocellulosic filler. The obtained results may indicate that the moisture absorption was inhibited by improving the interaction between the rPP matrix and the activated wood dust microparticles at the interface in the presence of the biomass. Evidently, the biomass microparticles reduce the availability of the hydroxyl groups on the wood surface due to their blocking by the interaction with the amine groups being in the tails and loops on the biomass surface. On the other hand, the same amine groups could simultaneously form hydrogen bonds with the rPP polymer matrix containing oxygen-containing functional groups during the WPC processing. Such a behavior of the WWB could confirm its function as a compatibilizer which promotes interfacial adhesion in the composite samples.

For predicting or assessing the degree of hydrophobicity of polymer products, a concept such as surface free energy (SFE) is employed [65,66]. The SFE values for the obtained WPC samples were calculated using the Owens–Wendt–Rabel–Kaelble (OWRK) approach. This approach considers surface tension in terms of polar and dispersed components. In such a manner, the energy of the surface layer of a solid has to involve two components, namely dispersive and polar. The dispersive component includes Van der Waals forces and other nonspecific interactions, and the polar component includes strong interactions and hydrogen bonds.

**Figure 13:**
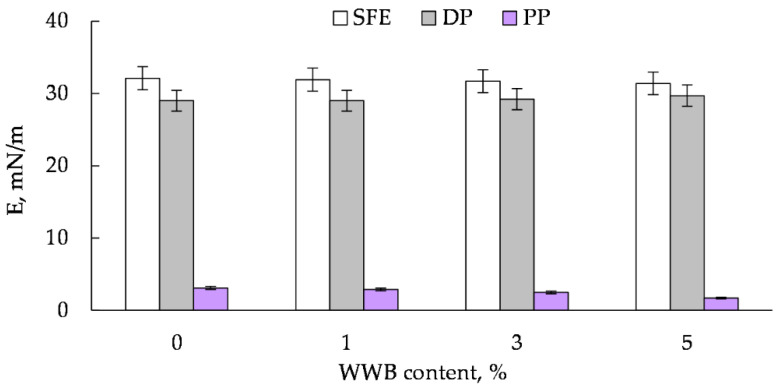
Surface free energy (SFE), its polar (PP) and dispersive (DP) parts of the wood–plastic composite sample with different contents of waste wood biomass.

Considering the major chemical and structural diversity of solids, three different liquids should be considered for characterizing new materials of unknown physicochemical properties, which significantly differ in terms of polar and dispersive interactions. Water, dimethyl sulfoxide and diiodomethane serve as the measured liquids in this study. The measurement results of the total surface free energy (SFE) for the WPC samples are given in Figure 13.

It can be seen that the biomass-free sample has the highest values of SFE and its polar part, namely 31.2 mN/m and 3.1 mN/m, respectively, while its dispersive part is characterized by the lowest value −29.0 mN/m at a 30% filling. With increasing the WWB content from 1% to 5% in the sample with the activated wood dust, the polar part of the SFE decreases essentially from 3.1 mN/m to 1.7 mN/m (approximately by 83%), while the drop in the total free energy and the enhancement of its dispersive part values are not significant. The remarkable decrease in the SFE polar part for the WPC sample with the growing hybrid filler content could be explained by the increase in its hydrophobicity due to the improvement of the interfacial adhesion between the rPP matrix and the activated wood particles in the presence of the WWB, performing the function of a bio-based compatibilizer. It should be noted that the total SFE and its dispersive part of the WPC samples containing the hybrid filler change relatively little in the presence of the biomass. This may be due to the 70% content of the hydrophobic polymer in the samples, which makes the main contribution to the values of these indicators.

**Figure 14:**
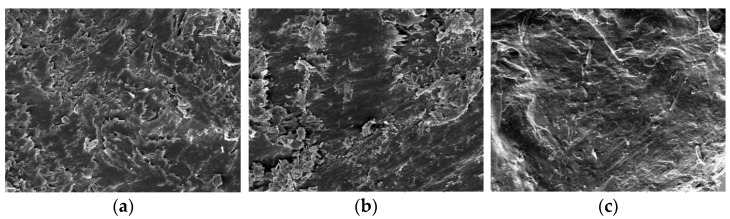
SEM images of the cross-section of the composite samples (×500) containing (**a**) activated wood dust, (**b**) activated wood dust with 1% and (**c**) 5% waste wood biomass.

Scanning electron microscope images of the obtained rPP-based composite samples are shown in Figure 14.

The morphology study of the cross-sections of the WPC sample filled with the activated wood sandling dust (Figure 14a) shows the non-uniform surface with the presence of many microdefects on it. On the cross-section of the sample, containing 1% of the biomass (Figure 14b), the quantity of the microdefects is fewer, but its surface is far from homogeneity. The comparison of the SEM cross-section images of the composite’s surfaces indicates a much bigger homogeneity and the lack of surface defects for the composite sample that contains the hybrid filler composed of 25% of the activated birch wood dust and 5% of the WWB microparticles (Figure 14c) compared to the case of other samples.

## 4. Conclusions

The wood wastewater biomass (WWB), a solid wastewater sludge formed as a result of model wastewater treatment with a new hybrid coagulant, representing a polymer complex of polyaluminium chloride with polyethylenimine, was used together with wood sanding dust to obtain the hybrid lignocellulosic filler in a quantity of 1% to 10% in terms of the rPP-based WPC weight. It was found that the WWB composed of high-molecular lignin and hemicelluloses substances was thermally stable and contained mainly particles ≤ 10 μk. The presence of the WWB in the hybrid filler led to an increase in mechanical properties, a decrease in water uptake and dimensional swelling, as well as a decrease in the polarity of the composite sample surface, while the content of the WWB in the samples did not exceed 5%. With further increasing the WWB content, the properties of the composite deteriorated. Such a behavior of the WWB indicated its ability to carry out the compatibilizer function. The coagulated biomass was suggested to improve the interface adhesion in the composite system due to the presence of free functional groups located in the biomass coagulate segments (tails, loops) that can interact with the surfaces of both the lignocellulosic filler and the rPP matrix during the composite processing.

## Figures and Tables

**Table 1:** Parameters of model wastewater.

pH	Biomass Content, mg L^−1^	Density, g cm^−3^	Chemical Oxygen Demand, mg L^−1^	Permanganate Index, mg L^−1^	Color, mgPt L^−1^
9.0–9.1	1400 ± 67	0.998	1285 ± 30	320 ± 10	746 ± 19

**Table 2:** Compositions of the studied wood–plastic composite samples.

Nr	Activated Wood Dust, %	Waste Wood Biomass, %	Recycled Polypropylene, %
1.	30	0	70
2.	29	1	70
3.	27	3	70
4.	25	5	70
5.	23	7	70
6.	20	10	70

**Table 3:** Elemental composition of the (a) evaporated and (b) coagulated waste wood biomass samples.

	C, %	H, %	O, %	N, %	Al, %	Cl, %
evaporated (a)	37.8	4.8	56.7	0.3	-	-
coagulated (b)	32.9	4.7	54.3	4.2	2.7	0.8

**Table 4:** FT-IR peaks assignments for evaporated and coagulated waste wood biomass samples.

Peak Positions (cm^−1^)	Peak Assignments	
	Evaporated	Coagulated
3433	Phenolic and aliphatic OH stretching	Phenolic and aliphatic OH stretching, N–H stretching
3289, 3186	C–H stretching in the low-molecular wood thermal hydrolysis products	
2937, 2860	C–H stretching in methoxyl, methyl and methylene groups	
1700, 1640, 1559	C=O stretching in ketones, carbonyls and ester groups	
1596		complex vibrations of C=O stretching bonded with N–H bending
1416	aromatic skeleton vibrations	
1331	syringyl ring breathing with C=O stretching	
1125–800	Vibration typical for hemicelluloses (C–C ring vibrations, overlapped with the stretching vibrations of C–OH side groups and the C–O–C glycosidic band vibrations)	

**Table 5:** Mechanical properties (tensile, bending) of wood–plastic composite samples with the hybrid filler.

Waste Wood Biomass Content, %	Tensile Strength, MPa	Young’s Modulus, MPa	Tensile Deformation, %	Bending Strength, MPa	Bending Modulus, MPa	Bending Deformation, mm
0 (initial dust)	20.9 ± 0.4	670.2 ± 6.5	15.6 ± 1.5	21.5 ± 0.7	1395 ± 25.5	9.6 ± 0.5
0 (activated dust)	27.7 ± 0.5	830.1 ± 6.8	12.7 ± 1.7	30.6 ± 0.9	2091 ± 26.7	8.1 ± 0.4
1	28.7 ± 0.7	849.8 ± 7.3	12.1 ± 1.9	31.3 ± 0.9	2140 ± 28.9	7.9 ± 0.7
3	29.3 ± 0.3	880.5 ± 7.7	11.0 ± 1.8	33.4 ± 0.7	2291 ± 26.9	7.3 ± 0.6
5	32.4 ± 0.3	950.7 ± 6.6	9.8 ± 1.5	37.1 ± 0.6	2540 ± 24.9	6.6 ± 0.5
10	28.1 ± 0.8	835.5 ± 9.1	11.9 ± 2.1	31.6 ± 0.9	2196 ± 29.1	7.7 ± 0.8

## Data Availability

Not applicable.
